# Identifying Hot-Spots of Metal Contamination in Campus Dust of Xi’an, China

**DOI:** 10.3390/ijerph13060555

**Published:** 2016-06-03

**Authors:** Hao Chen, Xinwei Lu, Tianning Gao, Yuyu Chang

**Affiliations:** School of Tourism and Environment, Shaanxi Normal University, Xi’an 710062, China; chenhao8848@126.com (H.C.); tianning_gao@sina.com (T.G.); changyuyu@snnu.edu.cn (Y.C.)

**Keywords:** metals, dust, hotspot, spatial cluster, Local Moran’s I

## Abstract

The concentrations of heavy metals (As, Ba, Co, Cr, Cu, Mn, Ni, Pb, V, and Zn) in campus dust from kindergartens, elementary schools, middle schools, and universities in the city of Xi’an, China, were determined by X-ray fluorescence spectrometry. The pollution levels and hotspots of metals were analyzed using a geoaccumulation index and Local Moran’s I, an indicator of spatial association, respectively. The dust samples from the campuses had metal concentrations higher than background levels, especially for Pb, Zn, Co, Cu, Cr, and Ba. The pollution assessment indicated that the campus dusts were not contaminated with As, Mn, Ni, or V, were moderately or not contaminated with Ba and Cr and were moderately to strongly contaminated with Co, Cu, Pb, and Zn. Local Moran’s I analysis detected the locations of spatial clusters and outliers and indicated that the pollution with these 10 metals occurred in significant high-high spatial clusters, low-high, or even high-low spatial outliers. As, Cu, Mn, Ni, Pb, V, and Zn had important high-high patterns in the center of Xi’an. The western and southwestern regions of the study area, *i.e.*, areas of old and high-tech industries, have strongly contributed to the Co content in the campus dust.

## 1. Introduction

Atmospheric pollution is a major hazard in urban environments, and atmospheric fallout plays an important role in the transport and fate of air pollutants. Atmospheric pollution is very serious in many densely populated cities, especially those with rapid industrialization and urbanization that have poor air quality and heavy depositions of dust [1,2,3,4]. Dust, which contains trace metals [5,6], accumulates in streets, gardens, schools, and residential areas with which people have frequent contact [7]. Dust particulates have been well recognized to influence human health [8,9,10], particularly due to their high contents of metals [11,12,13,14].

We previously studied the geochemical features of campus dust in the city of Xi’an in China [15] by analyzing databases containing data for many samples of dust from the campuses of a variety of educational institutions. Urban dust is spatially heterogeneous, so identifying the spatial patterns and hotspots of pollution in campus dust is difficult. A geographical information system (GIS) and multivariate analyses are useful for identifying spatial patterns of pollution and possible pollution sources [16,17]. Geostatistics is a widely used analytical method in GIS [18,19,20,21], but it cannot discriminate between positive or negative spatial correlations, detect spatial outliers or identify values that differ from those nearby. Local Moran’s I index, a local test statistic for spatial autocorrelation, is more widely used than geostatistics to identify spatial clusters and outliers of a variable [22,23,24]. It examines individual locations and identifies hotspots based on comparisons with neighboring samples [25]. The Local Moran’s I index has been successfully applied in the identification of hotspots of diseases [26,27], mortality rates [28,29], and in the environmental sciences [30,31].

Campuses contain some of the few playgrounds that students visit daily. Despite the serious effects of pollution on health, especially for children and young adults, studies in these areas are lacking, and information for metal contamination in academic urban campuses is limited. We assessed the levels of metal pollutants in dust sampled from a wide range of campuses in Xi’an, including kindergartens, elementary schools, middle schools, and universities. Our main objectives were to determine the concentrations of As, Ba, Co, Cr, Cu, Mn, Ni, Pb, V, and Zn (metals potentially harmful to the environment and human health) in campus dust, identify the pollution hotspots of heavy metals in the dust using Local Moran’s I and GIS and determine the local spatial patterns of heavy metals in the dust and the possible reasons for their formation.

## 2. Materials and Methods

### 2.1. The Study Area

Xi’an is the capital of Shaanxi Province and the largest city in northwestern China. It is in the center of the Guanzhong Plain (108°52′–108°60′E and 34°13′–34°19′N) in a topographic basin surrounded by the Qinling Mountains to the south and the Loess Plateau to the north. The Loess Plateau is the major source of Asian dust [32], which is heavily contaminated with airborne particulate matter (PM), especially in spring when dust storms are frequent [33]. The climate of Xi’an city is a typical temperate continental semi-humid climate with the annual average temperature and precipitation of 13–15 °C and 500–700 mm, respectively. The predominant wind direction in winter and autumn is northeast, while in summer and spring it is southwest [34]. Xi’an was established more than 2000 years ago, but rapid industrialization, urbanization and high-tech development in recent decades have pressured the city to adopt environmental protections and to repair environmental damage [35]. Xi’an had a total urban area of 3580 km^2^ and a population of 8,069,300 in 2013. The number of motorized vehicles has grown from 0.52 million in 2004 to 1.86 million in 2013 [36]. Xi’an contained 1295 kindergartens, 1291 elementary schools, 440 middle schools, and 63 universities in 2013, with 805,000 nursery and primary-school students, 484,000 middle-school students, and 838,000 university students [36].

### 2.2. Dust Sampling and Analysis

**Figure 1:**
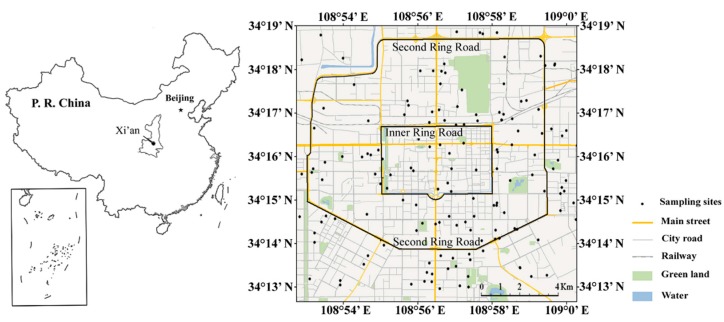
Maps indicating the location of Xi’an and the sampling sites in Xi’an.

We collected 157 dust samples from the nurseries, primary schools, middle schools, and universities using a clean plastic dustpan and brush [37,38,39] from windowsills, balconies, doorsteps, and playgrounds that were most accessible to the students (Figure 1). The samples were collected from each campus during the same dry season from October 2011 to October 2012 and were sealed in polyethylene bags for transport and storage. A Global Positioning System (GPS) was used to identify the locations of the sites of the dust samples. Each sample was air-dried in the laboratory for two weeks, sieved through a 1.0 mm nylon mesh to remove refuse and small stones and then divided into two subsamples.

All sieved samples for the geochemical analysis were finely ground to pass through a 200 (0.075 mm) mesh sieve to meet the requirements for X-ray fluorescence (XRF). Each 4.0 g milled dust sample and 2.0 g of boric acid were placed in a mold and pressed into 32-mm diameter pellet under 30 t of pressure. As, Ba, Co, Cr, Cu, Mn, Ni, Pb, V, and Zn concentrations were determined using a PANalytical PW2403 XRF analyzer, the relative proportions of dust were determined according to methods [6,40,41,42]. Table 1 listed the determination parameters and measuring conditions. The Chinese national reference materials GSS-1 and GSD12 [6], with an analytical precision >5%, were used for accuracy control. The repeatability of the measurements was confirmed by analyzing separate aliquots of 25% of the total sample set.

### 2.3. Outlier Detection

Environmental geochemical data sets often contain outliers [43] that should be detected and removed or replaced before statistical analysis. Many methods can detect outliers. The commonly used range method identifies outliers as those lower than the average values minus three standard deviations and those higher the average values plus three standard deviations. We deleted the outliers and replaced them with the highest values in the data sets [43].

### 2.4. Geoaccumulation Analysis

The geoaccumulation index (*I_geo_*) developed by Müller [44] is widely used to assess the level of heavy-metal contamination in dust [12,33,35,45,46]. *I_geo_* was calculated following Müller [44] as:

(1) $$I_{geo}=\log_{2}(C_{n}/(1.5B_{n}))$$

where *C_n_* is the concentration of heavy metal *n* in the dust sample and *B_n_* is the corresponding background concentration of heavy metal *n* in Shaanxi soil [47]. *I_geo_* consists of the following classifications [33,35,48]: uncontaminated (*I_geo_* ≤ 0), uncontaminated to moderately contaminated (0 < *I_geo_* ≤ 1), moderately contaminated (1 < *I_geo_* ≤ 2), moderately to heavily contaminated (2 < *I_geo_* ≤ 3), heavily contaminated (3 < *I_geo_* ≤ 4), heavily to extremely contaminated (4 < *I_geo_* ≤ 5), and extremely contaminated (*I_geo_* > 5).

### 2.5. Local Spatial Autocorrelation

Local Moran’s I can identify the autocorrelation between a single location and its neighbors [49]. It is calculated as:

(2) $$I_{i}=\frac{n(x_{i}-\bar{x})\sum_{j=1}^{n}w_{ij}(x_{j}-\bar{x})}{\sum_{i=1}^{n}{\left(x_{i}-\bar{x}\right)}^{2}}$$

where *n* is the number of observations of the entire region, *x_i_* is the value of variable *x* at location *i*, *x_j_* is the value of variable *x* at all other locations (where *j* ≠ *i*), $\bar{x}$ is the mean of *x*, and *w_ij_*, an element of spatial-weight matrix *w*, is the spatial weight between locations *i* and *j*.

A map showing locations with significant Local Moran’s I statistics, classified by type of spatial correlation, is defined as a cluster map of local indicators of spatial autocorrelation (LISA) [22]. Four categories of local spatial autocorrelation are distinguished: two suggest clusters that include high-high spatial clusters (high values in a high-value neighborhood) and low-low spatial clusters (low values in a low-value neighborhood) and two suggest spatial outliers that include low-high spatial outliers (a low value in a high-value neighborhood) and high-low spatial outliers (a high value in a low-value neighborhood). The LISA map provides information for the statistically significant clusters/outliers and indicates the variability and distribution of the types of spatial correlations in the study area. For dust pollution, low-low spatial clusters are “cool spots”, and high-high spatial clusters can be regarded as “regional hotspots”.

### 2.6. Data Transformation

Means and variances are strongly affected by positively skewed data (with some very high values), so data transformation was necessary before Local Moran’s I could be calculated. Box–Cox transformation is a common and effective method of power transformation [43,50,51,52]. The Box–Cox transformation is given by:

(3) $$y=\{\begin{array}{cc} \frac{x^{\lambda }-1}{\lambda } & \lambda \neq 0\\ \ln(x) & \lambda =0 \end{array}$$

where *y* is the transformed value and *x* is the value to be transformed. For a given data set (*x*_1_, *x*_2_, … *x_n_*), the parameter *λ* is estimated based on the assumption that the transformed values (*y*_1_, *y*_2_, … *y_n_*) are normally distributed. The transformation becomes logarithmic when *λ* = 0.

### 2.7. Statistical Analysis

Principal component analyses (PCA) are widely used to reduce data and to extract a small number of latent factors (principal components, PCs) for analyzing the relationships among observed variables [30,53]. The PCs are calculated based on a correlation matrix. Varimax with Kaiser normalization was used as the rotation method in the analysis [54]. A PCA can reduce the number of correlated variables to a smaller set of orthogonal factors, simplifying the interpretation of a given multidimensional system by displaying the correlations among the original variables [5].

### 2.8. Data Computation

All maps were produced using ArcGIS (version 9.3) (Esri, RedLands, CA, USA). Thiessen polygons of the samples were created, and spatial clusters/outliers were identified using Geoda (version 0.95i) (Arizona State University, Phoenix, AZ, USA) [55]. Statistical analyses were performed with SPSS 19.0 for Windows (IBM, Armonk, NY, USA).

## 3. Results and Discussion

### 3.1. Metal Concentrations in the Campus Dust

**Figure 2:**
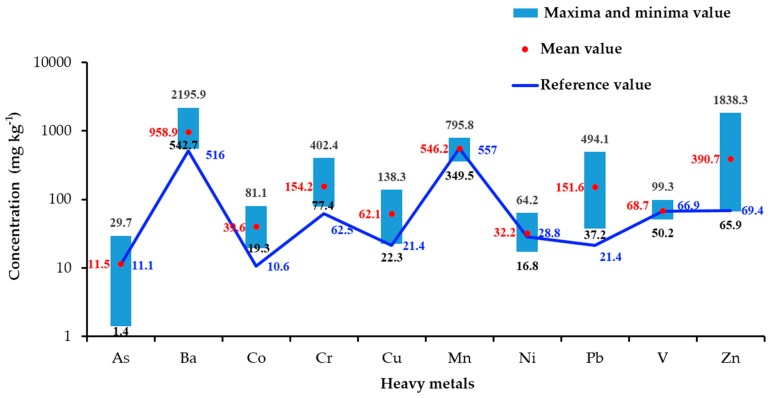
Metal concentrations in the campus dust in Xi’an and reference concentrations.

The metal concentrations in the campus dust from Xi’an are shown in Figure 2. The concentrations of As, Ba, Co, Cr, Cu, Mn, Ni, Pb, V, and Zn were within the ranges 1.4–29.7, 542.7–2195.9, 19.3–81.1, 77.4–402.4, 22.3–138.3, 349.5–795.8, 16.8–64.2, 37.2–494.1, 50.2–99.3, and 65.9–1838.3 mg·kg^−1^, respectively. The arithmetic mean concentrations of all analyzed metals in the dust were higher than their corresponding background concentrations in Shaanxi soil [47], except for As, Mn, Ni, and V. The coefficients of variation (CVs) were large for all heavy metals except Mn (17%), Ni (26%), and V (15%), indicating that the variations in the concentrations of As (50%), Ba (35%), Co (34%), Cr (41%), Cu (40%), Pb (61%), and Zn (77%) were high. The ratios of arithmetic mean concentrations of the heavy metals in the dust to the corresponding background concentrations in Shaanxi soil decreased in the order Pb > Zn > Co > Cu > Cr > Ba > Ni > As > V > Mn.

The ranges and mean concentrations of Cu, Pb, Zn, Cr, and Mn were lower in the campus dust than Xi’an road dust [33], perhaps because most schools are far from the main streets and because road dust is much more easily polluted than campus dust. The concentrations of As were similar in the campus and road dust [33].

Cu, Pb, and Zn were the most widely analyzed metals in the dust. Table 2 compares the Cu, Pb, and Zn concentrations in our samples with those from campuses in other cities [56,57,58,59,60,61]. The levels of Cu and Zn were higher in Xi’an than the other cities, except for Tehran and Hong Kong. The mean concentration of Pb was higher in Xi’an (151.6 mg·kg^−1^) than in Hermosillo (36.15 mg·kg^−1^), Shah Alam (31.24 mg·kg^−1^), and Beijing (69.4 mg·kg^−1^) but lower than in Tehran (257.4 mg·kg^−1^), Hong Kong (200 mg·kg^−1^), and Kaifeng (243 mg·kg^−1^).

### 3.2. Geoaccumulation Index Assessment (I_geo_)

**Figure 3:**
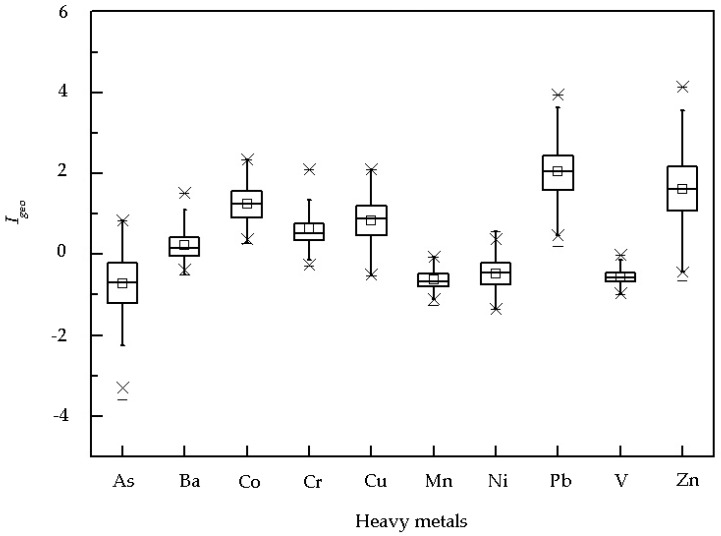
Geoaccumulation indices (*I_geo_*) of metals in the campus dust.

*I_geo_* was calculated for all analyzed metals in each dust sample relative to the background concentrations in the local soil [47] (Figure 3). *I_geo_* ranged from −3.57 to 0.83 for As, −0.51 to 1.5 for Ba, 0.28 to 2.35 for Co, −0.28 to 2.10 for Cr, −0.53 to 2.11 for Cu, −1.26 to −0.07 for Mn, −1.36 to 0.57 for Ni, 0.21 to 3.94 for Pb, −1.00 to −0.02 for V, and −0.66 to 4.11 for Zn. *I_geo_* averaged −0.71, 0.24, 1.24, 0.63, 0.84, −0.63, −0.47, 2.04, −0.56, and 1.62 for As, Ba, Co, Cr, Cu, Mn, Ni, Pb, V, and Zn, respectively. Mean *I_geo_* was >2 for Pb and <0 for As, Mn, Ni, and V. Maximum *I_geo_* was >2 for Co, Cr, and Cu, near 4 for Pb and >4 for Zn, indicating that the sources of Co, Cr, Cu, Pb, and Zn in the dust, especially Pb and Zn, were mostly associated with human activity. The *I_geo_* values of Ba in 31% and 62% dust samples were <0 and in 0–1, respectively.

### 3.3. Effects of the Weight Function on Hotspot Identification

To investigate the effects of different distance bands on the results of Local Moran’s I, we established three distance bands: 1000, 2000, and 5000 m. When calculating the index for each location, we assigned a value of 1 to the weights for neighboring locations if the distances were within a band, otherwise the weights were assigned a value of 0. Our choice of the distance bands was arbitrary, because no specific criteria were available for determining optimal distances. The distances should generally not be shorter than the sampling interval (about 500 m in this study) and not longer than half the maximum distance between all sample pairs (about 6000 m in this study). A distance band of 2000 m was most reasonable based on the results of previous distance-band analyses of the spatial distribution of metals in urban dusts [16,17].

### 3.4. Local Indicators of Spatial Association (LISA)

The LISA analysis indicated the spatial variability in detail. The concentrations of heavy metals in the dust were obviously higher than their corresponding background concentrations in Shaanxi soil, but only As, Cu, and Ni had a significant spatial pattern (Table 3). The percentages of samples with significant spatial clusters (high-high or low-low) were 44.0% for Ni, 43.3% for As, 36.3% for Cu but only about 20% for Co, Mn, Pb, and V. The high-high pattern of most of the heavy metals dominated the overall spatial pattern. More than 10% of the samples of most metals were significant spatial outliers (low-high and high-low), and the low-high pattern was particularly apparent for Cr, Mn, Pb, and Zn (Table 3). These significant spatial patterns may indicate enrichment of the heavy metals in the campus dust.

The pollution statuses of the metals in the categories of local spatial patterns are presented in Table 4. Mn and V were not pollutants in any of the categories of spatial patterns. Only 4.46% of the samples containing Ni had a high-high spatial pattern. The samples with As pollution are 15.29% and the main pollution is the high-high spatial pattern (9.56%). Although the samples with Ba pollution are 57.96% in no significantly categories of spatial pattern, there was only 1.27% in high-high spatial pattern. Co, Cr, Cu, Pb, and Zn represented a relatively high percentage of the polluted samples (Table 4). Among these, all samples were polluted by Co and Pb, and more than 90% of the samples were polluted by Cr, Cu, and Zn. The high-high spatial clusters for Co, Cr, Cu, Pb, and Zn decreased in the order Cu (21.66%) > Pb (14.65%) > Co (9.55%) > Zn (6.37%) > Cr (3.18%).

### 3.5. Metal Hot-Spots in Campus Dust

**Figure 4:**
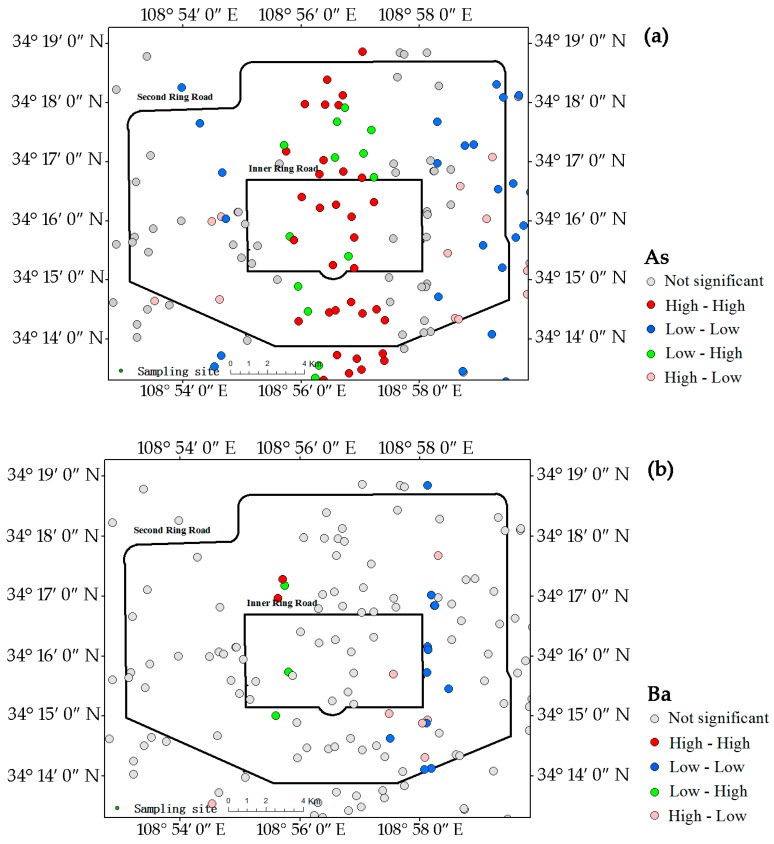

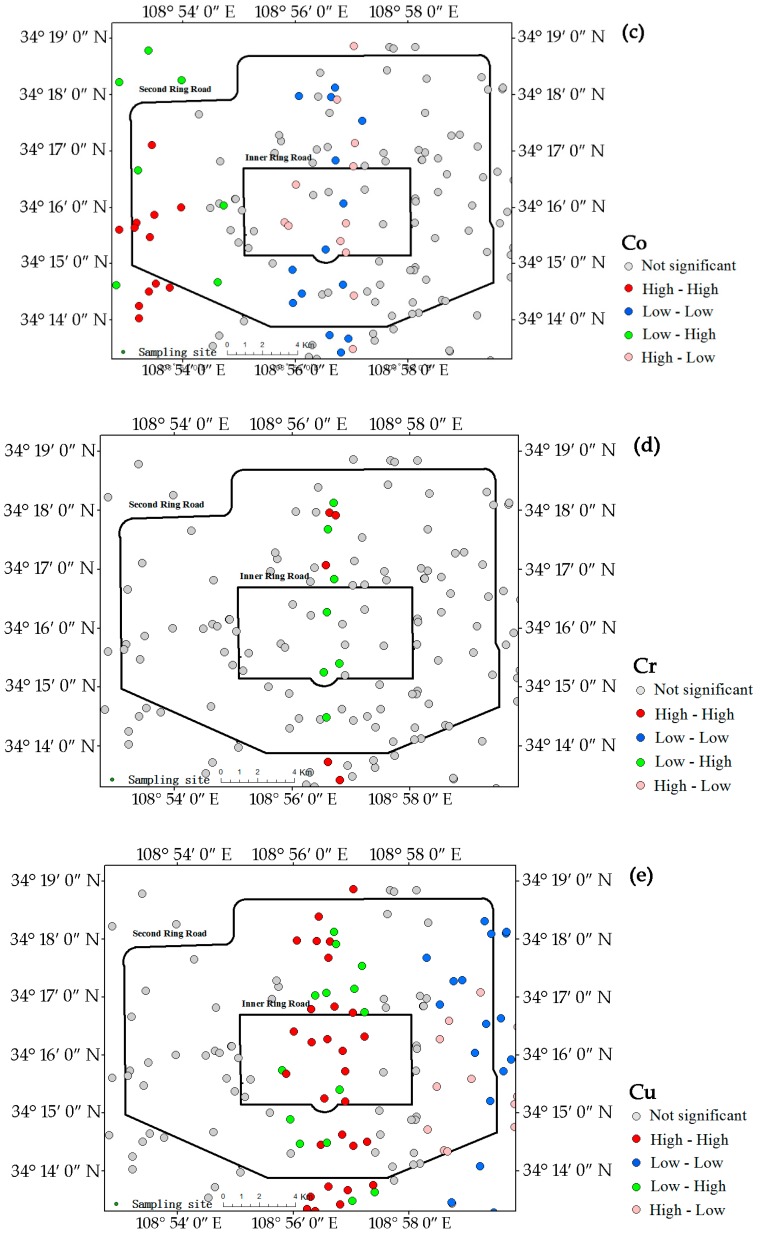

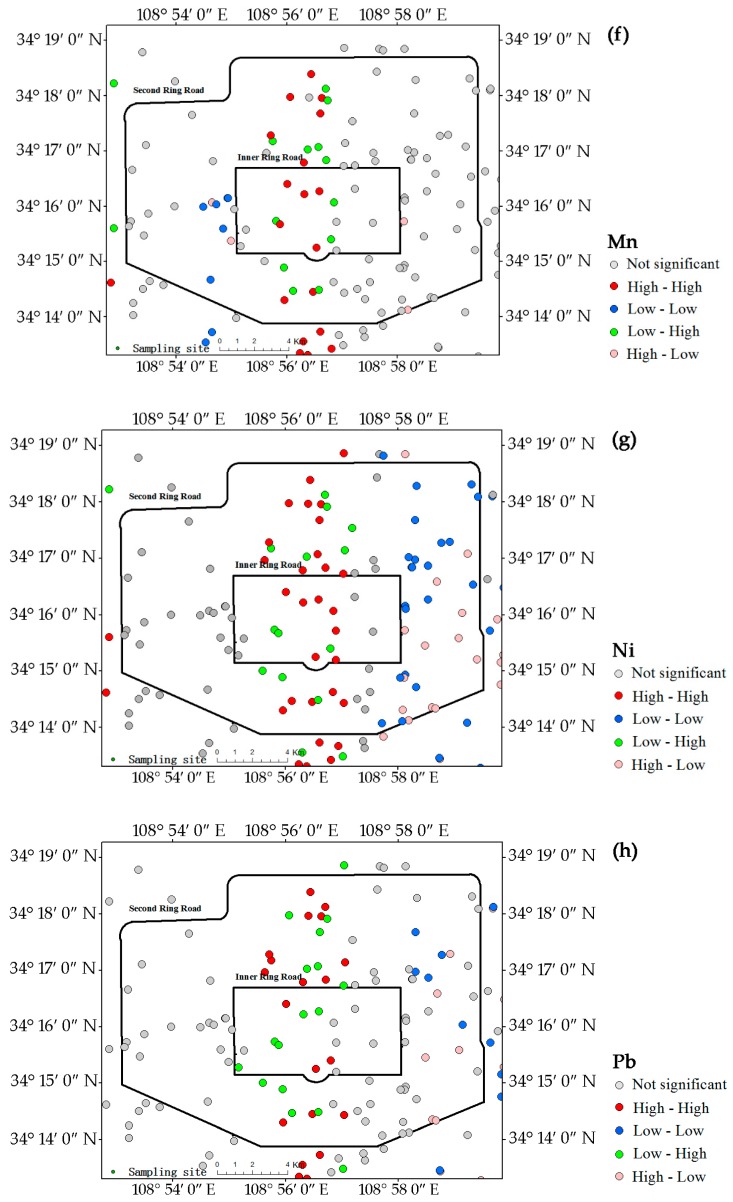

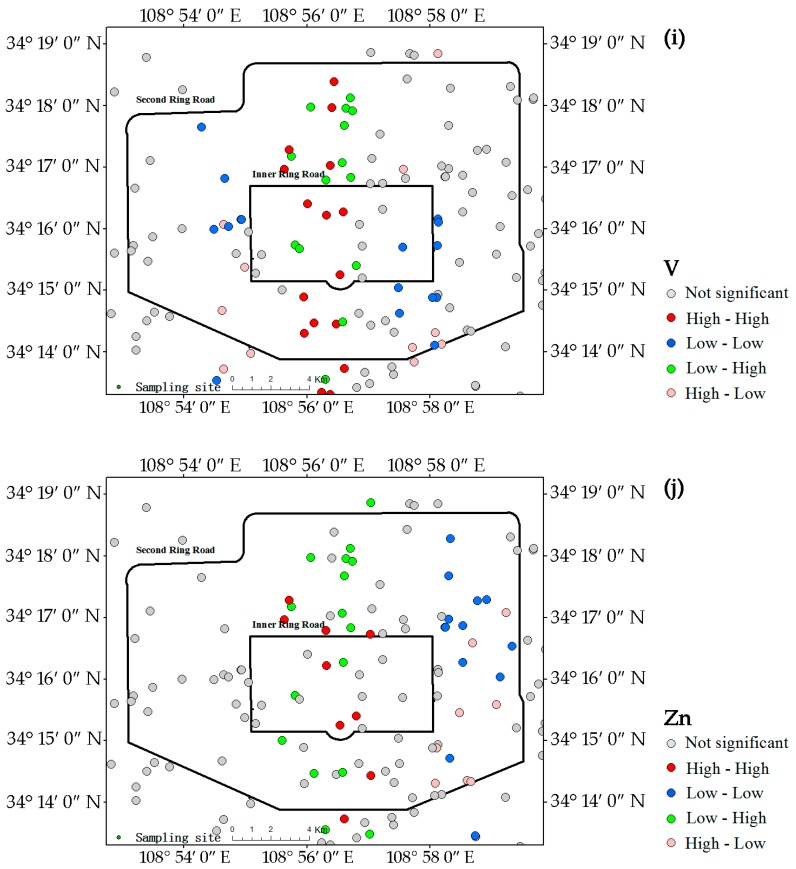
LISA cluster maps of Xi’an for metals in the campus dust. (**a**) As; (**b**) Ba; (**c**) Co; (**d**) Cr; (**e**) Cu; (**f**) Mn; (**g**) Ni; (**h**) Pb; (**i**) V; (**j**) Zn.

The LISA maps for all analyzed metals in the campus dust are presented in Figure 4, which further indicate the hot-spots of these metals. The central region extending from north to south was strongly influenced by the high-high pattern for As, Cu, Mn, Ni, Pb, and V, indicating that this zone contained most of the hot-spots of these metals. The central zone, which includes commercial centers and residential areas, has heavy traffic. The low-low patterns of As (Figure 4a) were evenly distributed in the western and eastern regions outside the inner ring road. Unlike As, the low-low patterns for Ni (Figure 4g), Cu (Figure 4e), and Pb (Figure 4h) were all concentrated in the eastern part of the study area, which contains an old industrial and commercial area. The numbers of low-low patterns of Ni, Cu, and Pb were notably lower in the east, and pollution by Ni, Cu, and Pb may be more intense in this area. The central region had fewer Mn (Figure 4f) and V (Figure 4i) hot–spots. The low-low patterns of Mn and V were sporadically distributed in the study area.

Unlike those of the other metals, the Co hot-spots (Figure 4c) were in the western and southwestern parts of the study area. The southwestern high-high pattern area was near the Second Ring road and contains a high-tech industrial area and the commercial district of Xi’an. The western high-high pattern corresponded to an old industrial area and a densely populated residential area. In contrast to the high-high patterns of most metals, the low-low pattern of Co appeared in the central region.

Ba (Figure 4b) and Cr (Figure 4d) had no obvious hot-spots, only a few low-low patterns of Ba in the eastern and southeastern parts of study area between the inner and second ring roads.

The Zn hot-spots (Figure 4j) were concentrated in the central region, similar to Pb, but in reduced number compared with Pb. The concentration, CV and *I_geo_* for Zn suggest that Zn concentration in dust is not only serious, but also generally prevalent.

### 3.6. Principal Component Analysis (PCA)

We conducted a PCA for each data set to gain insight into the sources of the metals and the major correlations among them. Table 5 shows the factor loadings with a varimax rotation and the eigenvalues and communalities calculated by SPSS. Five PCs had eigenvalues >1, and these five factors explained 80% of the total variance. The first factor explained 24.4% of the total variance, heavily loaded on As, Cu, Mn, and Ni. Factor 2 was loaded primarily by Ba and V and also moderately by Mn, accounting for 17.6% of the total variance. The loading was lower for Mn (0.442) than for V and Ba (0.878 and 0.814, respectively), which may imply quasi-independent behavior within the group [6]. Factor 3, dominated by Pb and Zn, accounted for 14.3% of the total variance. Factors 4 and 5 explained 12.5% and 11.2% of the total variance and loading for Cr and Co, respectively.

### 3.7. Causes of Hot-Spot Formation

The PCAs and the analysis of local spatial autocorrelation identified the main sources of hot-spot formation, which divided the metals into four groups. The first group contained As, Cu, Mn, Ni, Pb, V, and Zn, which had important high-high patterns and were strongly enriched in the central zone. Even though As, Mn, Ni, and V had many hot-spots in this area, the arithmetic mean concentrations of these metals in the dust samples were lower than or similar to their corresponding background levels. Mn, Ni, and V concentrations had low CVs, which may indicate that these metals in the central area originated naturally from the local soil. *I_geo_* indicated that As had mainly natural but partly anthropogenic sources. The concentrations of Cu, Pb, and Zn were >1.5-fold higher than the corresponding background concentrations in 95% of the samples. The Cu and Pb hot-spots were mainly concentrated in the central part of the city where the road network is dense and traffic is heavy. Cu is commonly used in Cu-brass automotive radiators due to its high resistance to corrosion and high thermal conductivity [62]. It is also often used in car lubricants [6]. The deterioration of the mechanical parts in vehicles over time will emit Cu to the surrounding environment [63], and the oxidation of lubricating oils upon exposure to air at high temperatures will form organic compounds that are corrosive to metals [64]. The use of leaded petrol has been banned in Xi’an since 2000, but the Pb content of the urban soil still represents substantial historical Pb contamination and the long half-life of Pb in soil [42]. Pb in bare soil could enter the urban dust by re-suspension. Zn was the second worst pollutant in the campus dust after Pb, but it had the fewest hot-spots of this first group of metals. The Zn hot-spots were masked by their contents, which increased in most regions. Zinc alloys and galvanized components are widely used in motor vehicles. Zinc compounds are also extensively used as antioxidants and as detergent/dispersant improvers for lubricating oils [65]. Zn added to tires during vulcanization accounts for 0.4%–4.3% of the tire tread [64]. The wear and tear of vulcanized vehicle tires and the corrosion of galvanized automobile parts are the main sources of Zn in urban environments [6,33].

The second group contained only Ba, with no obvious hot-spots in the study area. The low-low patterns of Ba were in the eastern and southeastern areas outside the inner ring road. The *I_geo_* assessment results indicated that Ba were uncontaminated and uncontaminated to moderately contaminated in the campus dust. Ba is widely used in alloys, paints, ceramics, plastic cements, and glass [66]. The characteristics of the spatial distribution of Ba concentrations in the dust suggest that the Ba originated mostly from natural sources but with some contribution from industrial emissions and construction.

The third group contained only Co. The CV of Co concentrations was relatively high, and Co concentrations in the urban dust were clearly higher than the corresponding background concentrations for Shaanxi soil, demonstrating that Co concentrations in the urban dust were mainly governed by human activity. The location of Co hot-spots, however, differed from those of the other metals, and the low-low patterns of Co were in the central zone, indicating that the sources of Co pollution differed from those of the first group of metals. The Co hot-spots were mainly in the southwestern area, which contains high-tech industries and many buildings. Co is extensively used in coating materials, paints, and pigments that are common in industrial products and modern buildings due to their gloss, faultless color, and visual impact. Co is thus prevalent in the southwestern high-tech industrial area, the hot-spot of Co in the campus dust.

The last group contained only Cr, whose concentrations were highly variable and were 2.5-fold higher than the corresponding background concentration. Cr concentrations in the dust were thus likely mainly due to human activity. Cr had few hot-spots north of the inner ring road and south of the second ring road, where traffic is heavy and population density is high. Cr is extensively used in automobile parts and aluminum and titanium alloys [16]. The characteristics of the distribution of Cr hot-spots suggest that the Cr in the campus dust originated mainly from automobile emissions and the combustion of fossil fuels.

## 4. Conclusions

The dusts from kindergarten, elementary school, middle school, and university campuses in Xi’an had elevated metal concentrations, especially those of Pb, Zn, Co, Cu, Cr, and Ba, which were 1.7–23.1, 0.9–26.5, 1.8–7.7, 1.0–6.5, 1.2–6.4, and 1.1–4.3-fold higher, respectively, than the corresponding background concentrations in Shaanxi soil. Mn, Ni, V, As, and Ba concentrations were generally low, and the Co, Cr, Cu, Pb, and Zn in the campus dust, especially Pb and Zn, were mostly due to human activity.

The distribution of hot-spots differed among the metals. As, Cu, Mn, Ni, Pb, V, and Zn had important high-high patterns in the center of the city. The Cu, Pb, and Zn hot-spots were strongly influenced by traffic. The high-high patterns of As, Mn, Ni, and V in the central zone were due to both human activity and natural factors. The Co hot-spots were mainly associated with industrial and construction activity. The Ba and Cr hot-spots were irregular in the Xi’an urban area. The concentrations and uses of the metals suggest that Ba originated mainly from natural sources but with some contribution from industrial emissions. Cr accumulated in the campus dust due to traffic and fossil-fuel combustion. The local governments should strengthen the monitoring and management of pollutants discharged from vehicles, fossil-fuel combustion, industrial products, and construction sites in the future.

## Figures and Tables

**Table 1:** Measuring condition of elements.

Channel	Type	Line	X-Tal.	Collimator (µm)	Detector	X-ray Tube Filter	Voltage (kV)	Current (mA)	Angle 2θ (°)
**As**	Gonio	Kα	LiF 200	300	SC	None	60	50	33.963
**Ba**	Gonio	Lα	LiF 200	300	FC	None	50	60	87.200
**Co**	Gonio	Kα	LiF 200	300	SC	None	60	50	52.792
**Cr**	Gonio	Kα	LiF 200	300	SC	None	60	50	69.368
**Cu**	Gonio	Kα	LiF 200	300	SC	None	60	50	45.035
**Mn**	Gonio	Kα	LiF 200	300	SC	None	60	50	62.982
**Ni**	Gonio	Kα	LiF 200	300	SC	None	60	50	48.663
**Pb**	Gonio	Lβ	LiF 200	300	SC	None	60	50	28.251
**V**	Gonio	Kα	LiF 200	300	FC	None	50	60	123.171
**Zn**	Gonio	Kα	LiF 200	300	SC	None	60	50	41.801

**Table 2:** Comparison of mean Cu, Pb, and Zn concentrations (mg·kg^−1^) in campus dust in various cities.

Element	Hermosillo	Tehran	Shah Alam	Hong Kong	Beijing	Kaifeng	Xi’an
**Cu**	26.34	225.3	30.19	247.38	57.3	38.92	62.1
**Pb**	36.15	257.4	31.24	199.96	69.4	242.99	151.6
**Zn**	387.98	873.2	148.71	2293.56	301	297.32	390.7

**Table 3:** Sample percentages (%) of the categories of local spatial patterns from the LISA analysis.

Spatial Autocorrelation	As	Ba	Co	Cr	Cu	Mn	Ni	Pb	V	Zn
**Not significant**	36.31	83.44	67.52	91.72	43.95	69.42	32.48	54.78	61.78	66.88
**High-high**	25.48	1.27	9.55	3.18	21.66	14.65	22.93	14.65	12.74	6.37
**Low-low**	17.83	7.64	8.92	0.00	14.65	4.46	21.02	9.55	9.55	9.55
**Low-high**	8.92	2.55	4.46	5.10	10.19	8.92	10.19	12.10	8.92	10.83
**High-low**	11.46	5.10	9.55	0.00	9.55	2.55	13.38	8.92	7.01	6.37

**Table 4:** The status of pollutant distribution (%) in the categories of local spatial patterns.

Heavy Metal	Pollution Status	Spatial Autocorrelation				
		Not Significant	High-High	Low-Low	Low-High	High-Low
**As**	Polluted	3.82	9.56	-	-	1.91
	Unpolluted	32.49	15.92	17.83	8.92	9.55
**Ba**	Polluted	57.96	1.27	3.18	1.27	5.10
	Unpolluted	25.48		4.46	1.28	-
**Co**	Polluted	67.52	9.55	8.92	4.46	9.55
	Unpolluted	-	-	-	-	-
**Cr**	Polluted	90.45	3.18	-	4.46	-
	Unpolluted	1.27	-	-	0.64	-
**Cu**	Polluted	43.95	21.66	12.1	8.28	9.55
	Unpolluted	-	-	2.55	1.91	
**Mn**	Polluted	-	-	-	-	-
	Unpolluted	69.42	14.65	4.46	8.92	2.55
**Ni**	Polluted	-	4.46	-	-	-
	Unpolluted	32.48	18.47	21.02	10.19	13.38
**Pb**	Polluted	54.78	14.65	9.55	12.1	8.92
	Unpolluted	-	-	-	-	-
**V**	Polluted	-	-	-	-	-
	Unpolluted	61.78	12.74	9.55	8.92	7.01
**Zn**	Polluted	65.61	6.37	8.92	10.19	6.37
	Unpolluted	1.27	-	0.63	0.64	-

**Table 5:** Rotated component matrix for the data of Xi’an campus dust (PCA loadings >0.4 are shown in bold).

Element	Component					Communalities
	1	2	3	4	5	
**As**	**0.845**	0.115	0.267	−0.117	0.007	0.812
**Ba**	0.015	**0.814**	0.334	0.114	0.220	0.837
**Co**	0.005	0.029	−0.022	0.010	**0.956**	0.915
**Cr**	0.169	0.023	0.024	**0.908**	0.008	0.855
**Cu**	**0.742**	0.004	0.102	0.345	0.057	0.759
**Mn**	**0.515**	**0.442**	0.102	0.386	−0.069	0.625
**Ni**	**0.816**	0.268	0.100	0.247	−0.070	0.813
**Pb**	0.269	0.132	**0.677**	0.249	−0.330	0.719
**V**	0.275	**0.878**	−0.010	−0.061	−0.135	0.868
**Zn**	0.260	0.149	**0.828**	−0.084	0.130	0.799
**Eigenvalue**	2.44	1.76	1.43	1.25	1.12	
**% of variance**	24.4	17.6	14.3	12.5	11.2	
**% of cumulative**	24.4	42.0	56.3	68.8	80.0	
